# Prediction of laparoscopic skills: objective learning curve analysis

**DOI:** 10.1007/s00464-022-09473-7

**Published:** 2022-08-04

**Authors:** A. Masie Rahimi, Sem F. Hardon, Ezgi Uluç, H. Jaap Bonjer, Freek Daams

**Affiliations:** 1 Department of Surgery, Amsterdam UMC – VU University Medical Center, Amsterdam, The Netherlands; 2 Amsterdam Skills Centre for Health Sciences, Tafelbergweg 47, 1105 BD Amsterdam, The Netherlands

**Keywords:** Laparoscopy, Training, Prediction of skill, Simulation, Learning curve, Minimally invasive surgery

## Abstract

**Introduction:**

Prediction of proficiency of laparoscopic skills is essential to establish personalized training programs. Objective assessment of laparoscopic skills has been validated in a laparoscopic box trainer with force, motion and time recognition. The aim of this study is to investigate whether acquiring proficiency of laparoscopic skills can be predicted based on performance in such a training box.

**Methods:**

Surgical residents in their first year of training performed six different tasks in the Lapron box trainer. Force, motion and time data, three objective measures of tissue manipulation and instrument handling, were collected and analyzed for the six different tasks. Linear regression tests were used to predict the learning curve and the number of repetitions required to reach proficiency.

**Results:**

A total of 6010 practice sessions performed by 42 trainees from 13 Dutch hospitals were assessed and included for analysis. Proficiency level was determined as a mean result of seven experts performing 42 trials. Learning curve graphs and prediction models for each task were calculated. A significant relationship between force, motion and time during six different tasks and prediction of proficiency was present in 17 out of 18 analyses.

**Conclusion:**

The learning curve of proficiency of laparoscopic skills can accurately be predicted after three repetitions of six tasks in a training box with force, path length and time recognition. This will facilitate personalized training programs in laparoscopic surgery.

**Supplementary Information:**

The online version contains supplementary material available at 10.1007/s00464-022-09473-7.

Surgeons require a specific set of advanced technical skills to safely perform minimally invasive surgery (MIS). These technical skills include hand–eye coordination, depth perception and handling long instruments with reduced tactile feedback [1–4]. The surgical residency program has followed an apprenticeship model of ‘‘see one, do one, teach one’’ for more than a century [5]. However, after the introduction of the relatively complex MIS, this approach of training in the operating room resulted in increased patient injuries and associated health care costs [6–8]. During the last decades, reports on improvement of patient safety by simulation training have been published [9–11]. This has started a paradigm shift of the way surgeons are trained, following a new model of “see one, simulate many, do one’’ [5, 12].

Traditionally, laparoscopic skills have been assessed subjectively using forms such as OSATS, GOALS, and OPRS [13–15]. However, it is important to include objective assessment in skills training to provide supervisors with a consistent tool to assess the skills of surgical residents [1]. A combination of performance parameters has been classified, representing tissue manipulation and instrument handling skills, which enables objective assessment of laparoscopic skills [16]. Our research group reported earlier successful implementation of objective performance parameters in basic laparoscopic skills training of first-year surgical residents, enabling objective assessment of learning curves [17].

Objective assessment of the learning curve is essential to determine when proficiency levels have been acquired [17–20]. It is demonstrated that baseline performances of psychomotor ability uniquely predict the learning curve during laparoscopic skills training with virtual reality simulators [21, 22]. Predicting learning curves at an early stage of training allow creating individually adjusted skills training programs in the near future [21, 23]. The aim of this study was to analyze and predict the learning curve of basic laparoscopic technical skills.

## Methods

### Participants

First-year surgical resident who completed a basic laparoscopy course between April 2020 and June 2021 were included for prospective data-analysis. Surgical residents from multiple teaching hospitals in the Netherlands were included. The Basic Laparoscopy Course is part of the surgical residency program and participating in the study was voluntary (and without consequences). The study was exempt from Ethical Board review.

### Protocol and the basic laparoscopy course

The basic laparoscopy course consisted of a 3-week at-home laparoscopic box training course, followed by a hands-on training day at the Amsterdam Skills Centre, consisting of performing a laparoscopic appendectomy and cholecystectomy on fix for life cadaver models [17]. Trainees received a laparoscopic box trainer and were instructed to train a minimum of five sessions a week, performing six different validated laparoscopic tasks [13, 24–26] (Supplemental File A). Measurements were compared to predefined proficiency levels, which were equal to mean parameter outcomes of 7 surgeons [13]. The scoring system consists of a scale of 1–10 with 8 being the proficiency level (pre-set competency based on experts). The score consists of the average of the force, motion and time which are each scored individually.

At the end of this course, the trainees performed the six tasks once again as a post-course assessment. Overall progression was measured by comparing baseline and post-course assessment. Objective force, motion and time parameters were measured, representing tissue manipulation and instrument handling skills [16, 27].

### System and materials

The Lapron box trainers (Amsterdam Skills Centre, Amsterdam, The Netherlands) [28] were utilized during the basic laparoscopy course. The box trainers were equipped with the ForceSense objective measuring system (MediShield B.V., Delft, the Netherlands) [29], which uploaded all measurements and recordings to an online database. Six previously validated laparoscopic tasks were included: Post and Sleeve, Loops and Wire, Flap task, Wire chaser, Pattern cut and Zigzag loop [17] (Supplemental File A). Furthermore, the Lapron box trainer was equipped with two curved Maryland grasping forceps, one laparoscopic scissor and a laparoscopic axial needle holder (Aesculap, B. Braun, Melsungen, Germany). All statistical analyses were performed using the 26th version of IBM SPSS Statistics. Graphs were created using GraphPad (Prism 9.0.0, San Diego, California USA).

### Statistical analyses

Learning curves that show maximum force (*N*), path length (mm) and time (*s*) were created for the six tasks, displaying the group mean and proficiency levels. The path length was defined as the total distance travelled by the laparoscopic instruments and the maximum force was defined as the maximum absolute force applied on the laparoscopic tasks [13].

Linear regression tests were performed to predict the learning curve at an early stage of training using IBM SPSS statistics 28 (SPSS Inc., Chicago, Illinois USA). Baseline performances of the parameters maximum force (*N*), path length (mm) and time (*s*), were included as independent variables. These baseline performances were defined as the average scores of the first three measurements. The number of sessions that were needed to reach the proficiency level were included as dependent variables. Linear regression tests were performed separately for each parameter of the six tasks. All sessions that were not successfully completed due to unforeseen circumstances and tasks that were performed less than three times were excluded from analysis. Trainees that did not reach the proficiency level for one of three parameters were excluded from analysis of this specific parameter. Post hoc power analyses were performed using GPower (Supplemental File A, Table A1).

## Results

### Learning curve analysis

**Fig. 1:**
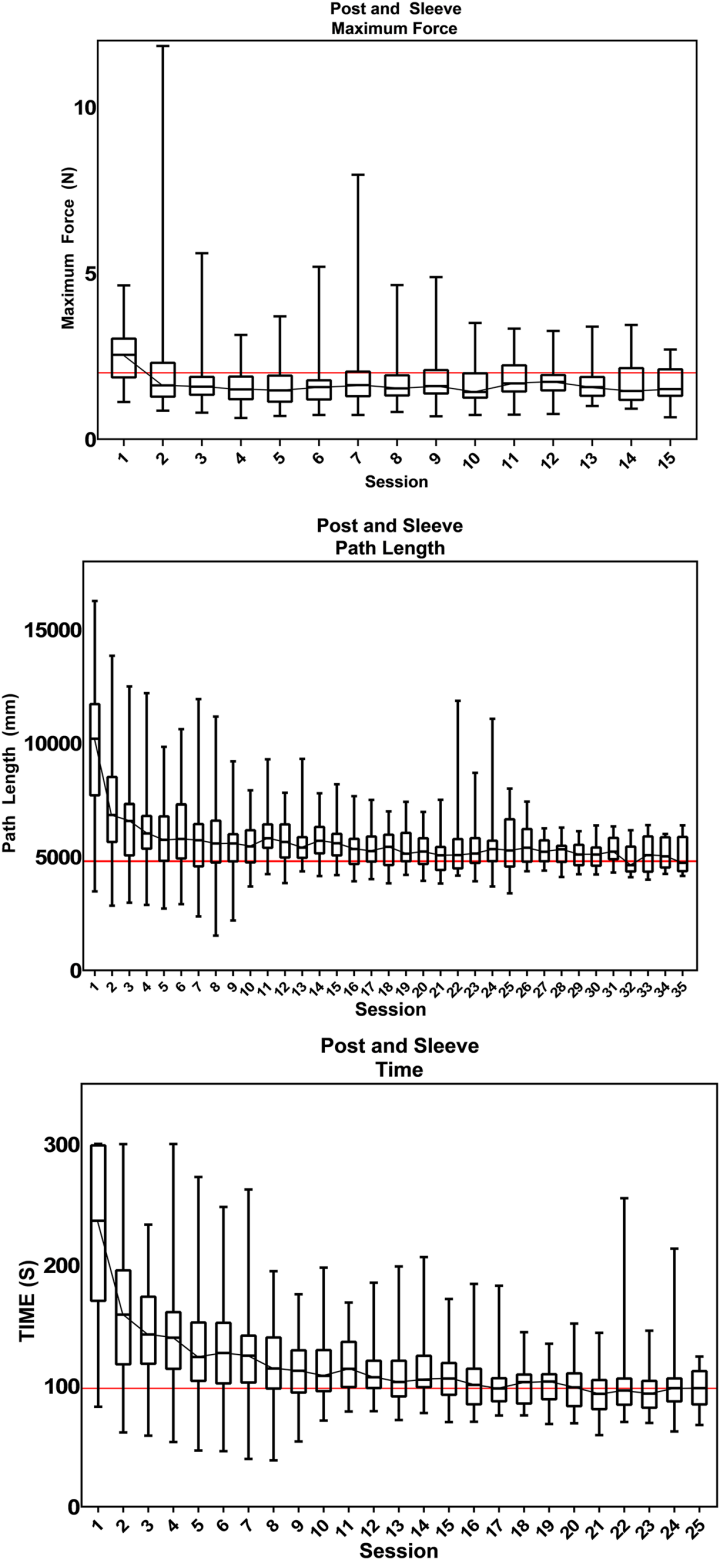
Post and Sleeve learning curves

A total of 6010 trials, performed by 42 trainees from 13 Dutch hospitals were included for analysis. Figure 1 shows the proficiency graphs of the parameters: maximum force (*N*), path length (mm) and time (*s*) for the Post and Sleeve laparoscopic task. Proficiency level graphs of all six tasks are provided in Supplemental file B.

For the Post and Sleeve, the benchmark of maximum force was reached at the 4th session, while the benchmark of the path length was reached at the 32nd session and the benchmark of time at the 21st session of the training. Supplementary Figures B2-B6 show an improvement of the mean and standard deviation over the initial training sessions, after which it gradually levels out in the plateau phase.

Table 1 shows the proficiency level of each parameter and the number of trainees that reached the proficiency level. For all tasks, the proficiency level of the maximum force was the first to be acquired, except for the Wire chaser, in which the benchmark for the maximum force was reached at the 38th session. The benchmarks of the parameters time and path length were reached at the same time for the Flap task and the Pattern cut. While for the other tasks, the proficiency level for time reached before the proficiency level of the path length. Moreover, 19 out of 42 reached proficiency for the path length of the Loops and wire, 21 out of 42 trainees reached the proficiency level of the maximum force for the Wire chaser, and 31 out of 42 trainees reached the proficiency level of the path length for the Zigzag loop. These three parameters were reached by the lowest number of trainees. The remaining 15 parameters were reached by more than three quarters of the trainees.

**Table 1:** Mean session to proficiency and number of trainees that reached the proficiency level

Task	Benchmark	Mean session to reach proficiency	Number of trainees that reach proficiency
Post and sleeve			
Time (*s*)	98.4 s	21	38/42
Path length (mm)	4810 mm	32	37/42
Maximum force (*N*)	2.00 N	4	42/42
Loops and wire			
Time (*s*)	86.0 s	18	38/42
Path length (mm)	3300 mm	35	19/42
Maximum force (*N*)	3.01 N	2	42/42
Flap task			
Time (*s*)	42.8 s	36	36/42
Path length (mm)	1993 mm	36	37/42
Maximum force (*N*)	1.56 N	2	42/42
Wire chaser			
Time (*s*)	106.6 s	6	40/42
Path length (mm)	4558 mm	19	37/42
Maximum force (*N*)	1.22 N	38	21/42
Pattern cut			
Time (*s*)	150.1 s	3	42/42
Path length (mm)	6000 mm	3	42/42
Maximum force (*N*)	2.22 N	1	40/42
Zig-zag loop			
Time (*s*)	54.6 s	30	35/42
Path length (mm)	3027 mm	44	31/42
Maximum force (*N*)	2.70 N	7	41/42

*Note* Mean session at which trainees reach the proficiency level is determined by proficiency graphs (Fig. 1 and Supplementary Fig. 2–6)

### Learning curve prediction

The results of the Linear regression analyses are provided in Table 2. For 17 of 18 parameters, the baseline performance had a statistically significant relationship with the number of sessions needed to reach the benchmark. Within the path length of the Loops and wire, this relation was insignificant. Fifteen out of 18 dependent variables were not normally distributed and therefore were either log-transformed or square-root transformed, as shown in Table 2. The relation between the number of sessions needed to reach the benchmark and the baseline performance was quadratic for two out of 18 parameters. For these parameters, curvilinear regression tests were performed in which squared independent variables were included for analysis.

**Table 2:** Results of linear regression analyses: baseline performances as a predictor of the number of sessions needed to reach the benchmark of the parameters time, path length and maximum force

		B	SE	t	Sig. (p)	95% CI
Post and sleeve						
Time	Constant	− 9.19	3.238	− 2.39	0.007 *	[− 15.756, − 2.621]
	X	0.12	0.018	6.801	0.000 *	[0.085, 0.156]
Path length**	Constant	− 0.17	0.175	− 0.95	0.348	[− 0.521, 0.189]
	X	1.29 E-4	0	5.823	0.000 *	[0.000, 0.000]
Maximum force**	Constant	0	0.093	− 0.01	0.996	[− 0.190, 0.189]
	X	0.188	0.04	4.646	0.000 *	[0.106, 0.270]
Loops and wire						
Time ***	Constant	0.602	0.63	0.955	0.346	[− 0.676, 1.881]
	X	0.016	0.004	3.588	0.001 *	[0.007, 0.025]
Path length **	Constant	0.705	0.375	1.882	0.077	[− 0.085, 1.496]
	X	5.38 E -5	0	0.86	**0.402 n.s**	[0.000, 0.000]
Maximum force **	Constant	− 0.4	0.087	− 4.58	0	[− 0.571, − 0.22]
	X	0.206	0.028	7.483	0.000 *	[0.150, 0.261]
Flap task						
Time **	Constant	− 0.11	0.318	− 0.34	0.74	[− 0.753, 0.541]
	X	0.015	0.005	2.684	0.011 *	[0.004, 0.026]
	X2 ****	− 4.21 E-5	0	− 2.03	0.050 *	[0.000, 0.000]
Path length **	Constant	0.230	0.12	1.915	0.064	[− 0.014, 0.474]
	X	1.12 E-4	0	4.718	0.000 *	[0.000, 0.000]
Maximum force **	Constant	− 0.31	0.07	− 4.47	0	[− 0.455, − 0.171]
	X	0.36	0.048	7.565	0.000 *	[0.263, 0456]
Wire chaser						
Time **	Constant	− 0.34	0.162	− 2.08	0.044	[− 0.665, − 0.009]
	X	0.006	0.001	5.334	0.000 *	[0.004, 0.008]
Path length **	Constant	− 2.25	0.754	− 2.99	0.005	[− 3.789, − 0.719]
	X	0.001	0	3.245	0.003 *	[0.000, 0.001]
	X2 ****	− 3.94 E-8	0	− 2.51	0.017 *	[0.000, 0.000]
Maximum force **	Constant	− 0.707	0.344	− 2.05	0.054	[− 1.428, 0.14]
	X	− 0.76	0.193	3.967	0.001 *	[0.361, 1.167]
Pattern cut						
Time **	Constant	− 0.51	0.111	− 4.57	0	[− 0.733, − 0.282]
	X	0.005	0.001	7.231	0.000 *	[0.004, 0.007]
Path length **	Constant	− 0.51	0.115	− 4.47	0	[− 0.747, − 0.280]
	X	1.13 E-4	0	7.392	0.000 *	[0.000, 0.000]
Maximum force **	Constant	− 0.29	0.071	− 4.08	0	[− 0.437, − 0.146]
	X	0.206	0.039	5.348	0.000 *	[0.128, 0.285]
Zig-zag loop						
Time	Constant	4.656	4.345	1.072	0.292	[− 4.183, 13.495]
	X	0.076	0.034	2.239	0.032 *	[0.007, 0.145]
Path length	Constant	1.712	5.212	0.329	0.745	[− 8.964, 12.389]
	X	0.002	0.001	2.76	0.010 *	[0.001, 0.004]
Maximum force **	Constant	− 0.19	0.144	− 1.32	0.195	[− 0.480, 0.101]
	X	0.155	0.041	3.809	0.000 *	[0.073, 0.238]

*Note* Estimates are unstandardized coefficients. *ns* not significant; *p ≤ 0.05; **Log-transformed (Log10) dependent variable; ***Square-root-transformed dependent variable; ****Squared independent variable. p ≤ 0.05 are given in bold

Table 3 shows the linear regression equations for the estimation of the number of sessions needed to reach the benchmark. Transformed models are either Log – Linear (Log10) or Square-root – Linear. Post hoc power-analysis revealed high power (> 0.8) for 16 out of 18 linear regression tests. The power of the path length within the Loops and wire was 0.138, while the power of time within the Zig-zag loop was 0.610. See Supplementary file A for results of the post hoc power-analyses (Supplementary Table A1).

**Table 3:** Learning curve Prediction: linear regression equations. *Y* = Number of sessions needed to reach the benchmark; *X* = Baseline performances of the parameters time, path length and maximum force

Task	Untransformed data	Transformed data
Post and sleeve		
Time	*Y* = − 9.188 + 0.120 * X	
Path length		Log10 (Y) = − 0.166 + 1.29 E-4 * X
Maximum force		Log10 (*Y*) = 0.000 + 0.118 * X
Loops and wire		
Time		SQRT (*Y*) = 0.602 + 0.016 * X
Path length	n.s	
Maximum force		Log10 (*Y*) = − 0.396 + 0.206 * X
Flap task		
Time		Log10 (*Y*) = − 0.106 + 0.015 * X – (− 4.21 E-5) * X2
Path length		Log10 (*Y*) = 0.230 + 1.12 E-4 * X
Maximum force		Log10 (*Y*) = − 0.313 + 0.360 * X
Wire chaser		
Time		Log10 (*Y*) = − 0.337 + 0.006 * X
Path length		Log10 (*Y*) = − 2.245 + 0.001 * X – (− 3.94 E-8) * X2
Maximum force		Log10 (*Y*) = − 0.707 + (− 0.764 * X)
Pattern Cut		
Time		Log10 (*Y*) = − 0.508 + 0.005 * X
Path length		Log10 (*Y*) = -0.514 + 1.13 E-4 * X
Maximum force		Log10 (*Y*) = − 0.292 + 0.206 * X
Zig-zag loop		
Time	*Y* = 4.656 + x * 0.076	
Path length	*Y* = 1.712 + x * 0.002	
Maximum force		Log10 (Y) = − 0.189 + 0.155 * X

*Note* Estimates are unstandardized coefficients. Transformed models are either Log – Linear (Log10) or Square-root – Linear. ns not significant

## Discussion

**Fig. 2:**
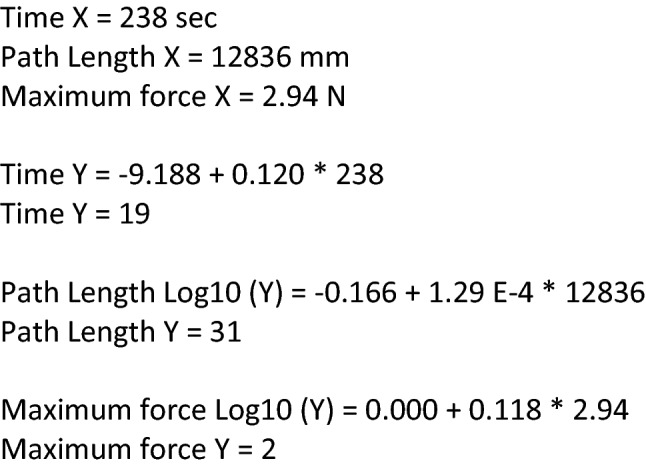
Example of prediction calculation

This study showed that it was possible to predict the learning curve of laparoscopic technical skill in a basic laparoscopy course at an early stage of training. By performing a laparoscopic task three times, it is possible to calculate how many repetitions are needed to acquire the benchmark for force, motion and time parameters. For example, according to the calculations in Fig. 2, 19 repetitions for reaching the time benchmark, 31 repetitions for reaching the path length benchmark and two repetitions to not exceed maximum force are advised when a trainee completed the Post and Sleeve task three times with the following average outcome: time 238 s, path length 12,836 mm and maximum force 2.94 N.

This allows identifying trainees who require more time and feedback for their laparoscopic training. Furthermore, the possibility arises to find trainees require less training time for basic laparoscopic skills, and hence, can advance earlier to more complex laparoscopy. Lastly, using the current methods it is possible to recreate this learning curve prediction model for other laparoscopic (and robotic) training tasks and curricula.

Proficiency graphs displaying the group learning curve made it possible to analyze the learning curve and determine if and when the proficiency level is reached for each parameter of the six tasks. For all except one tasks, the benchmark of maximum force was the first to be reached. The benchmarks of the parameters time and path length were either reached at once, or the benchmark of time was reached before the benchmark of the path length. This indicates that trainees need the most time to improve their path length. This is supported by the analysis of the number of trainees that reach the proficiency level, in which the benchmark of the path length was reached the least often. This is consistent with our prior conducted research [17]. At the start of the training, the majority of novices is focused on safe tissue manipulation and on completion of the task. Resulting in a longer completion time and more instrument movements. Furthermore, efficient handling of instruments is more inherent to experts and their proficiency levels in this metric are relatively high. Using the above mentioned prediction model, more feedback and guidance can now be given in an early phase for path length parameters.

Furthermore, differences between tasks and parameters can be examined. It is found that the proficiency levels for the Zig-zag loop are reached at a later stage of training, while the three benchmarks for the Pattern cut are reached within three sessions, suggesting that the Pattern cut is easier to perform, compared to other tasks.

The learning curve of all residents improved rapidly during the first sessions, after which it gradually leveled out in the plateau phase, which is as expected [30]. This suggests that it is possible to predict the learning curve at an early stage of training. Stefanidis et al. (2017) stated that baseline performances, which were defined as average scores of the first three measurements, might be of value in the prediction of skill acquisition in laparoscopic skills training with virtual reality simulators [31]. This was consistent with our analysis, the mean of the first three measurements were defined as baseline performances and included as predictor variables in the prediction of the learning curve in the basic laparoscopy course.

Improving a personalized curriculum could be achieved by showing the trainees performance level and the proficiency levels during training. This enables comparing the trainees performance with the proficiency level, the group mean and quartiles 1 to 3. Displaying these proficiency graphs during training can enhance the individual feedback that is received directly by trainees. [32]. As an example of personalized training, the Amsterdam UMC and the 13 affiliated teaching hospitals have a Minimally Invasive Surgery Curriculum in which personalized training is implemented. Since 2018, the Basic Laparoscopy Course is mandatory for junior residents. The course participants receive box training with objective feedback and are examined on fix4life human cadavers in the Amsterdam Skills Centre. After obtaining the certificate, the residents perform laparoscopic procedures in the OR.

A limitation of this study is that trainees that did not reach the proficiency level were excluded from analysis within the learning curve prediction. This could have the effect that the prediction model is more optimistic in prediction. This was because for statistical reasons making a prediction model only the trainees that achieved proficiency could be reliably used for prediction. However, the prediction model is still able to distinguish between underperformers and overperformers in an early phase. This implies that overperformers would not need to use expensive training facilities for an extended period. And underperformers will be identified in an early phase for additional personalized training. A personalized prediction model can be applied universally for all trainees.

In conclusion, measurement of objective force, motion and time parameters can predict the time of reaching proficiency allowing tailored and personalized training.

## Supplementary Information

Below is the link to the electronic supplementary material.

Supplementary file1 (DOCX 4702 KB)
