# Supplementary material for: Prediction of laparoscopic skills: objective learning curve analysis
Source: Surg Endosc. 2022 Aug 4;37(1):282–9. doi: 10.1007/s00464-022-09473-7 (PMC9839814; doi:10.1007/s00464-022-09473-7)
Supplement: Supplementary file 1 — Supplementary file1 (DOCX 4702 KB) [file 464_2022_9473_MOESM1_ESM.docx]

**Supplemental file A:**

*Basic Laparoscopy Course tasks and power analysis*

**Fig A1**

*The six laparoscopic tasks of the Lapron box trainer*


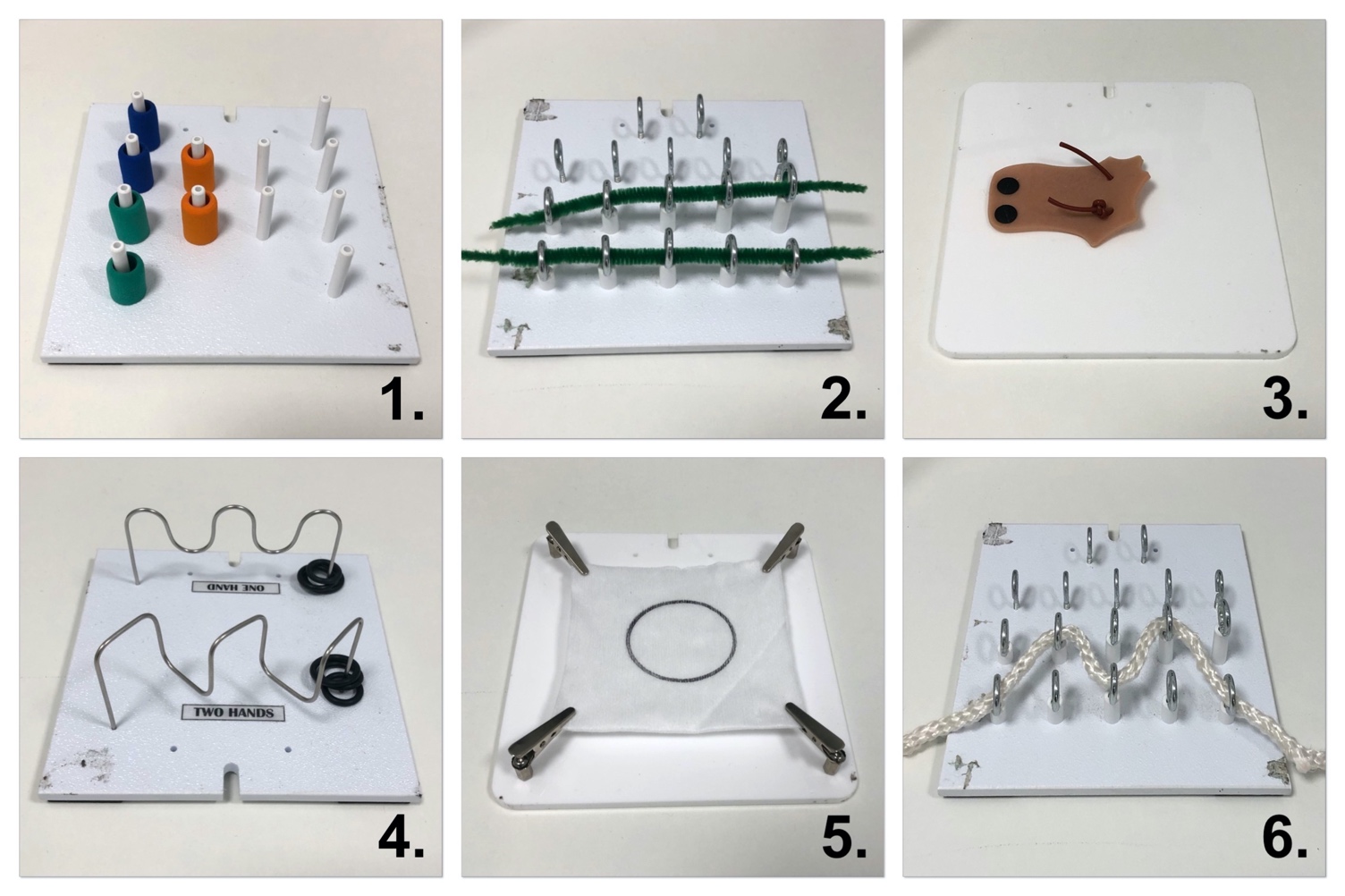


NB From upper left to bottom right the following tasks are displayed: 1. Post and Sleeve, 2. Loops and Wire, 3. Flap task, 4. Wire Chaser, 5. Pattern Cut and 6. Zig-zag Loop.

**Table A1**
*Post hoc power-analysis results*

| Task | R^2^ | Power |
| --- | --- | --- |
| Post and sleeve |  |  |
| Time  Path length  Maximum force | 0.562  0.492  0.362 | 0,999  0,999  0,997 |
| Loops and wire |  |  |
| Time  Path length  Maximum force | 0.263  0.042  0.577 | 0.948  0.138 *  1.000 |
| Flaptask |  |  |
| Time  Path length  Maximum force | 0.234  0.389  0.595 | 0.896  0.997  1.000 |
| Wire chaser |  |  |
| Time  Path length  Maximum force | 0.435  0.429  0.453 | 0.999  0.998  0.979 |
| Pattern Cut |  |  |
| Time  Path length  Maximum force | 0.599  0.603  0.450 | 1.000  1.000  0.999 |
| Zig-zag loop |  |  |
| Time  Path length  Maximum force | 0.132  0.214  0.276 | 0.610 *  0.801  0.971 |

*Note.* GPower was used for power-analysis. * = power < 0,8

**Supplemental file B:**

*Learning curve analysis of the six BLC tasks*


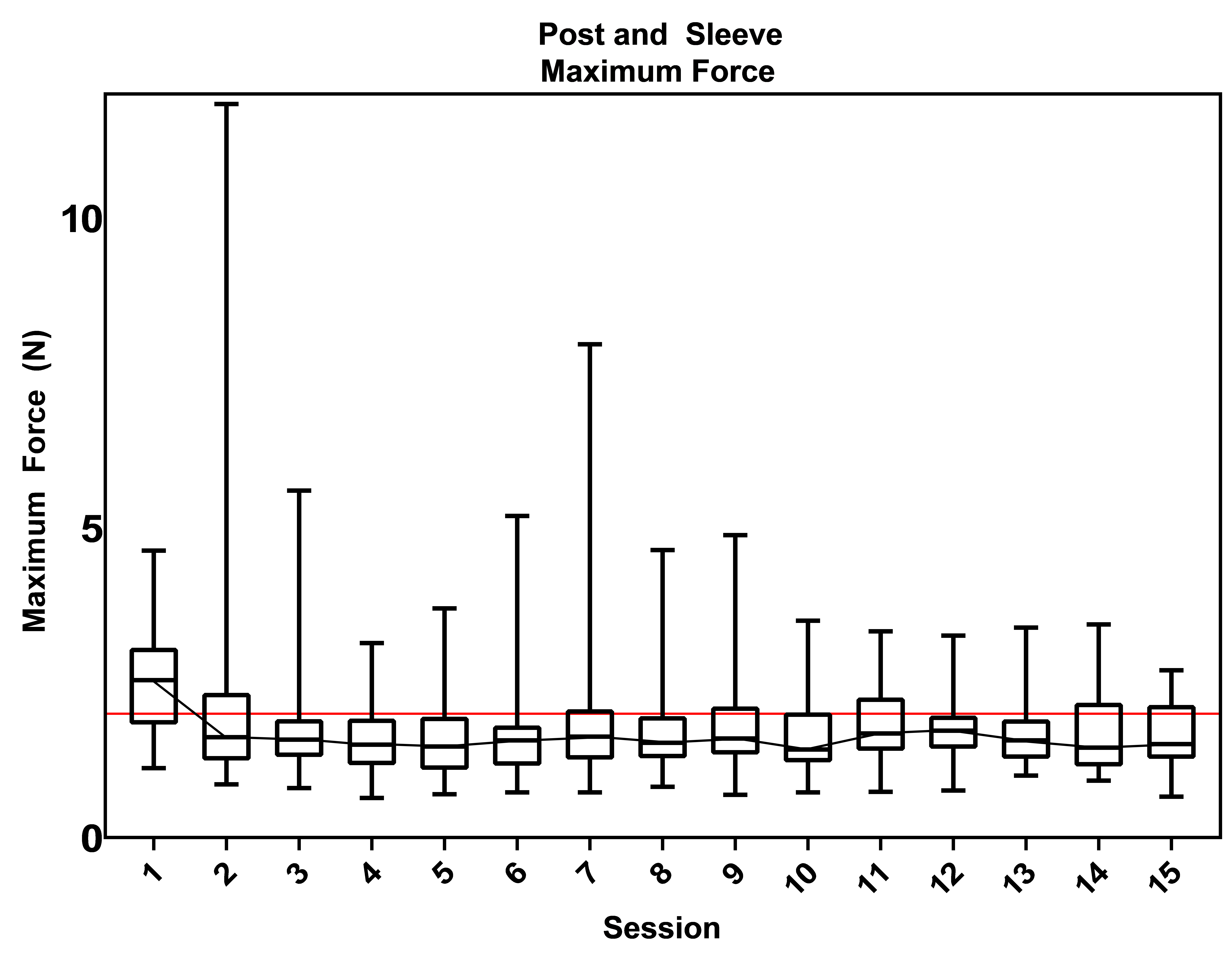


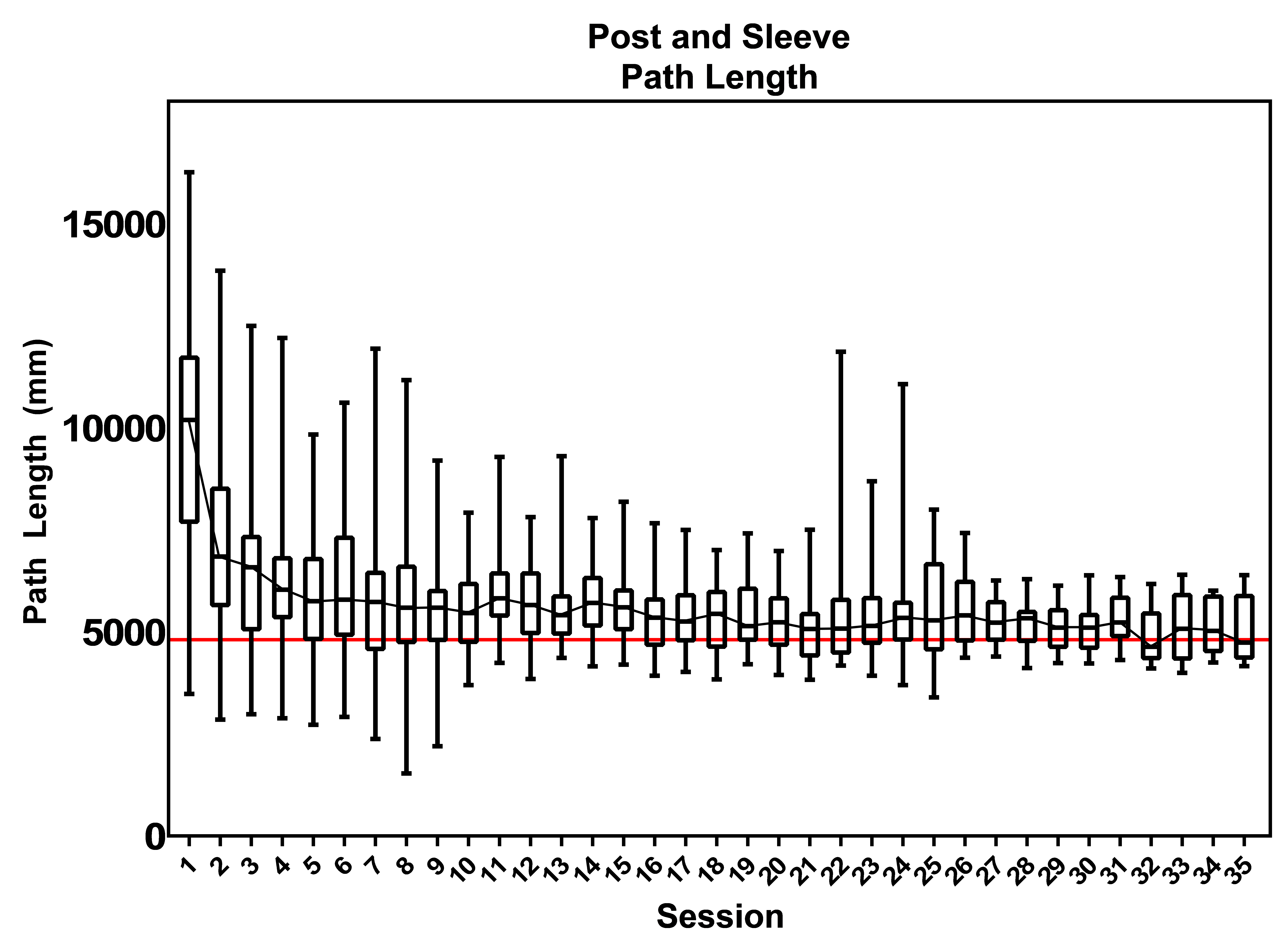


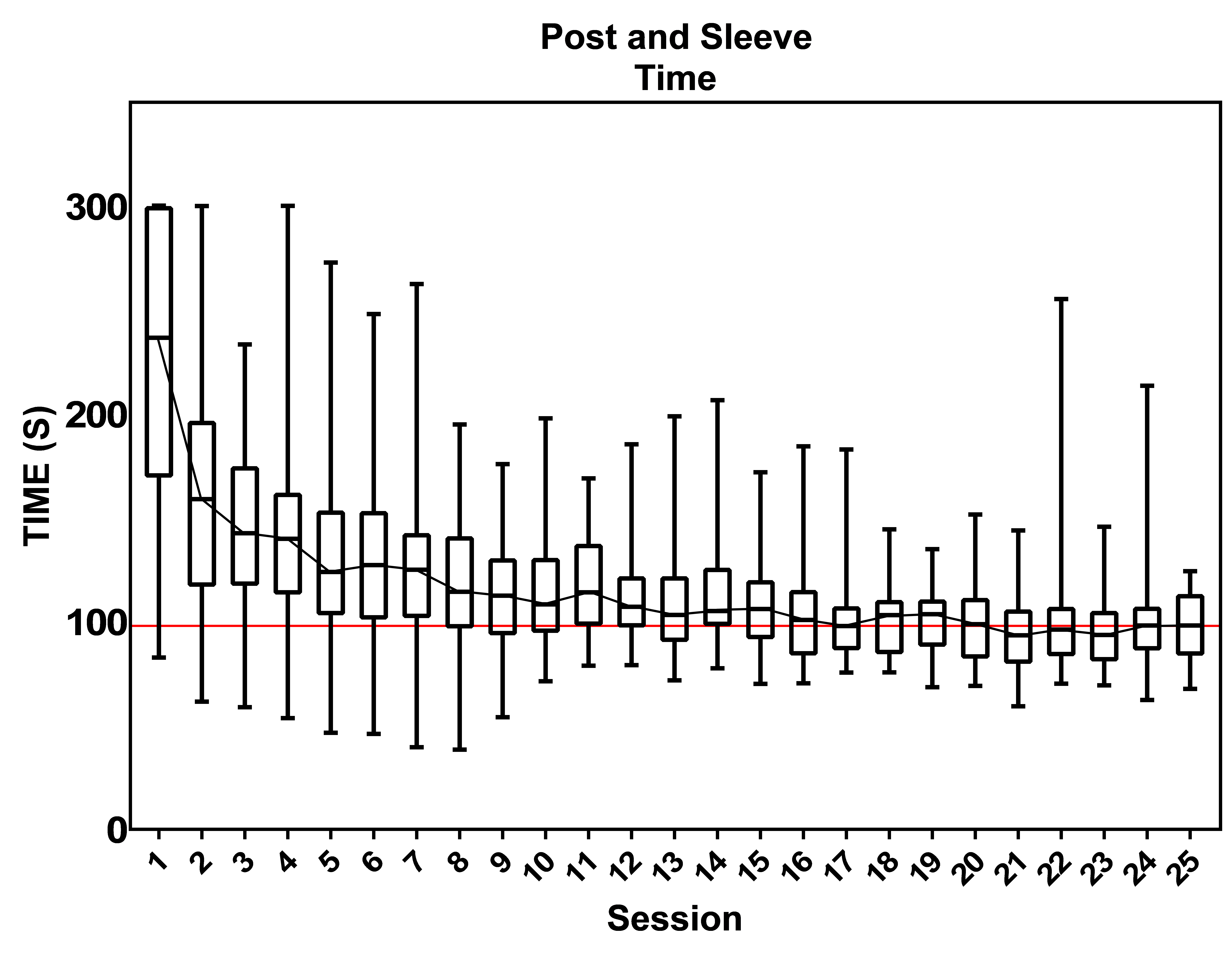


**Fig B1.** Proficiency graphs of task 1 “Post and sleeve”.


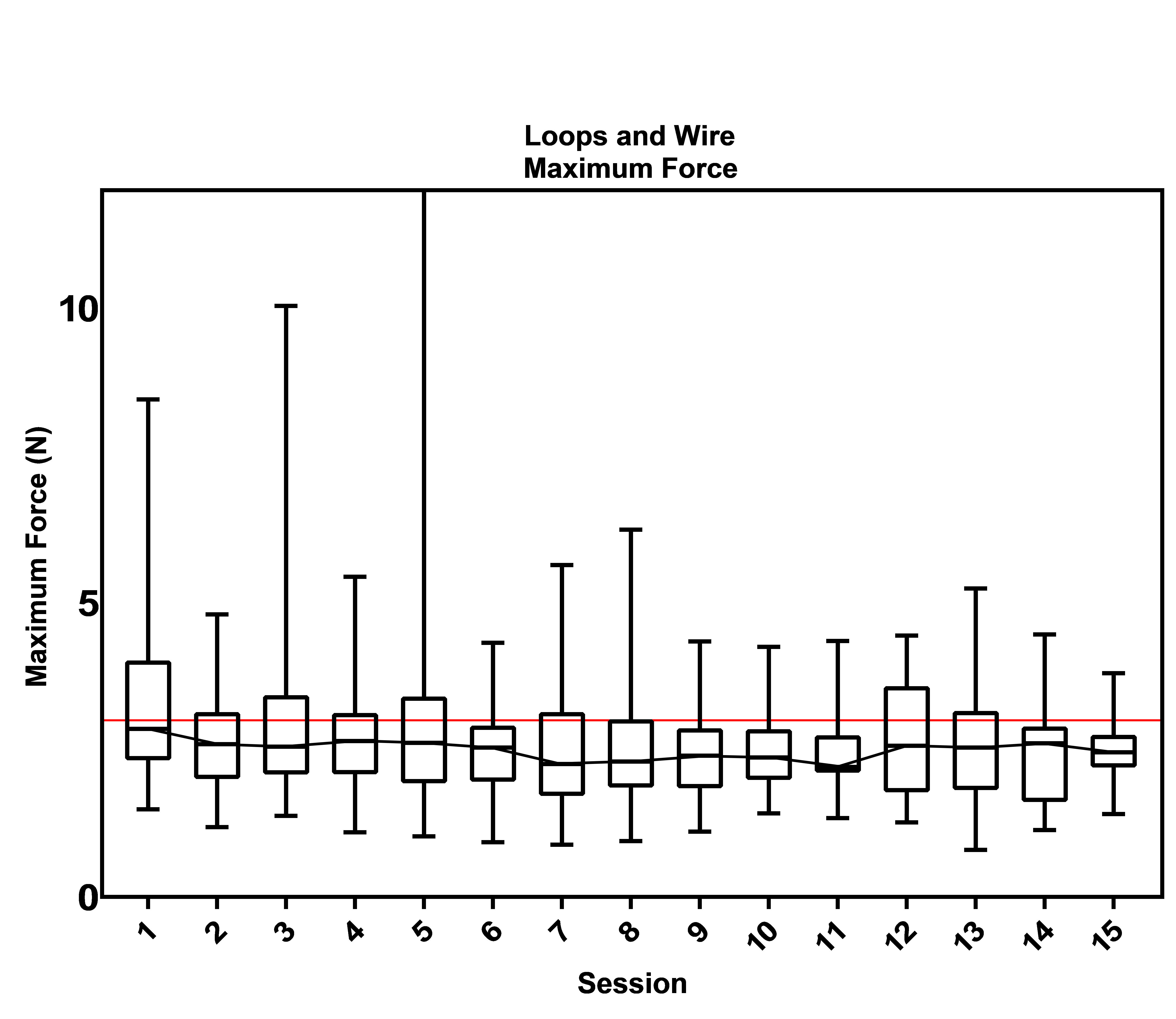


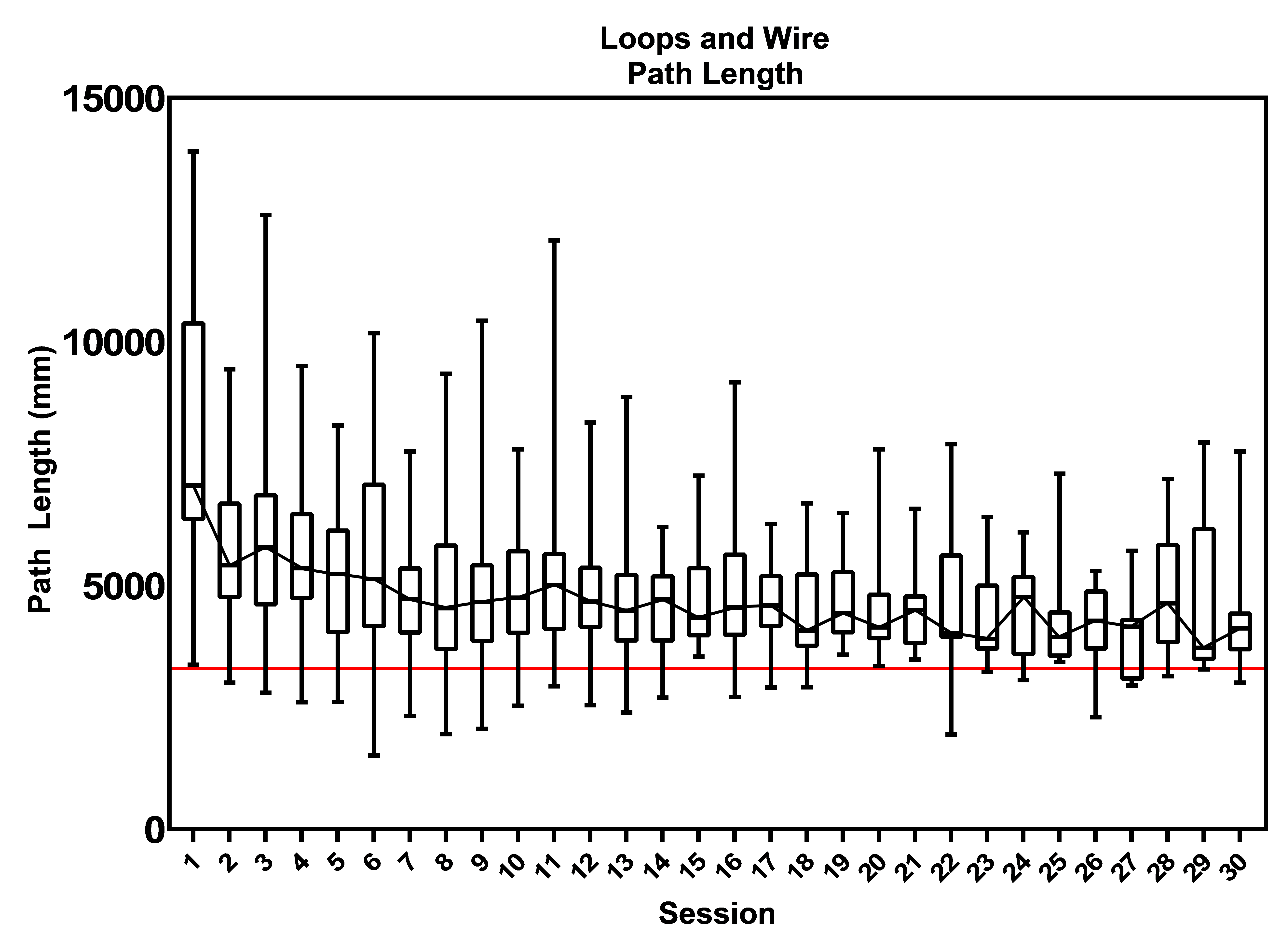


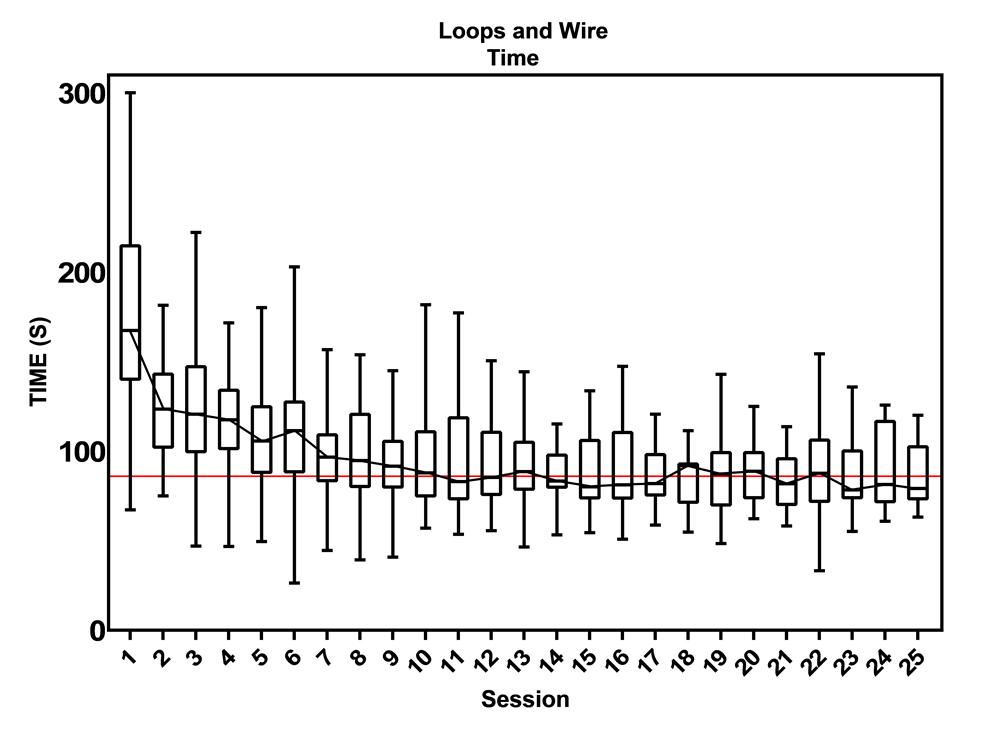


**Fig B2.** Proficiency graphs of task 2 “Loops and Wire”.


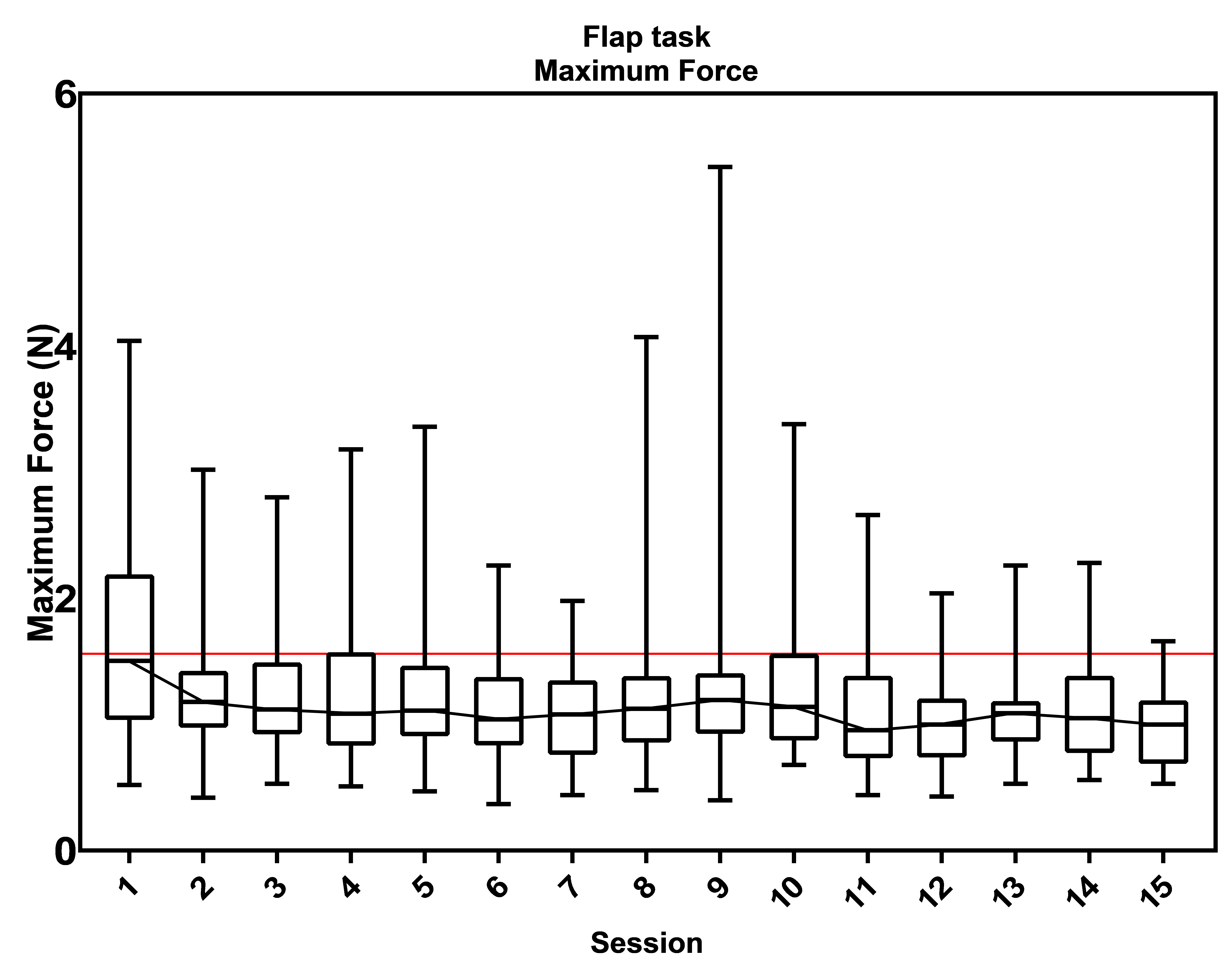


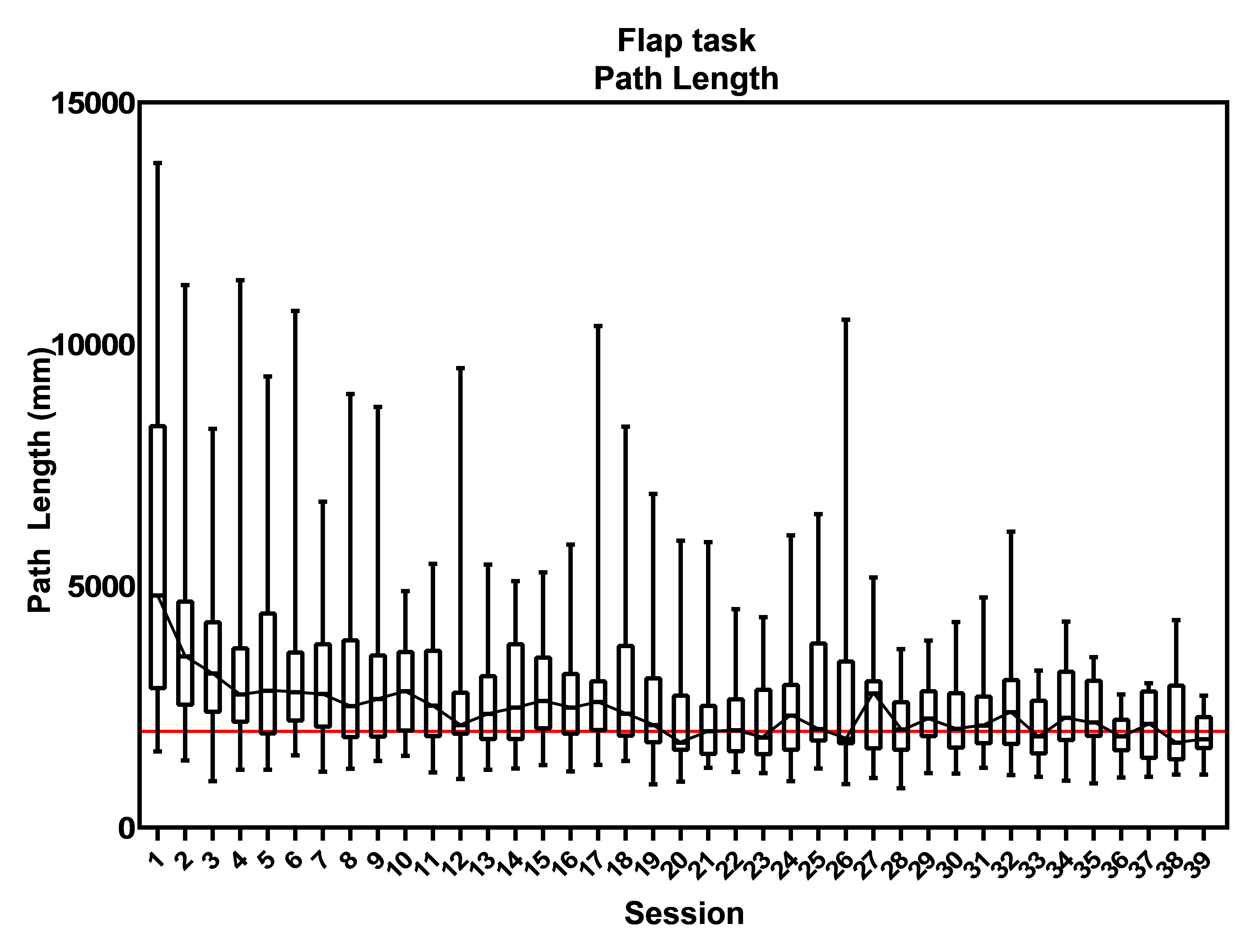


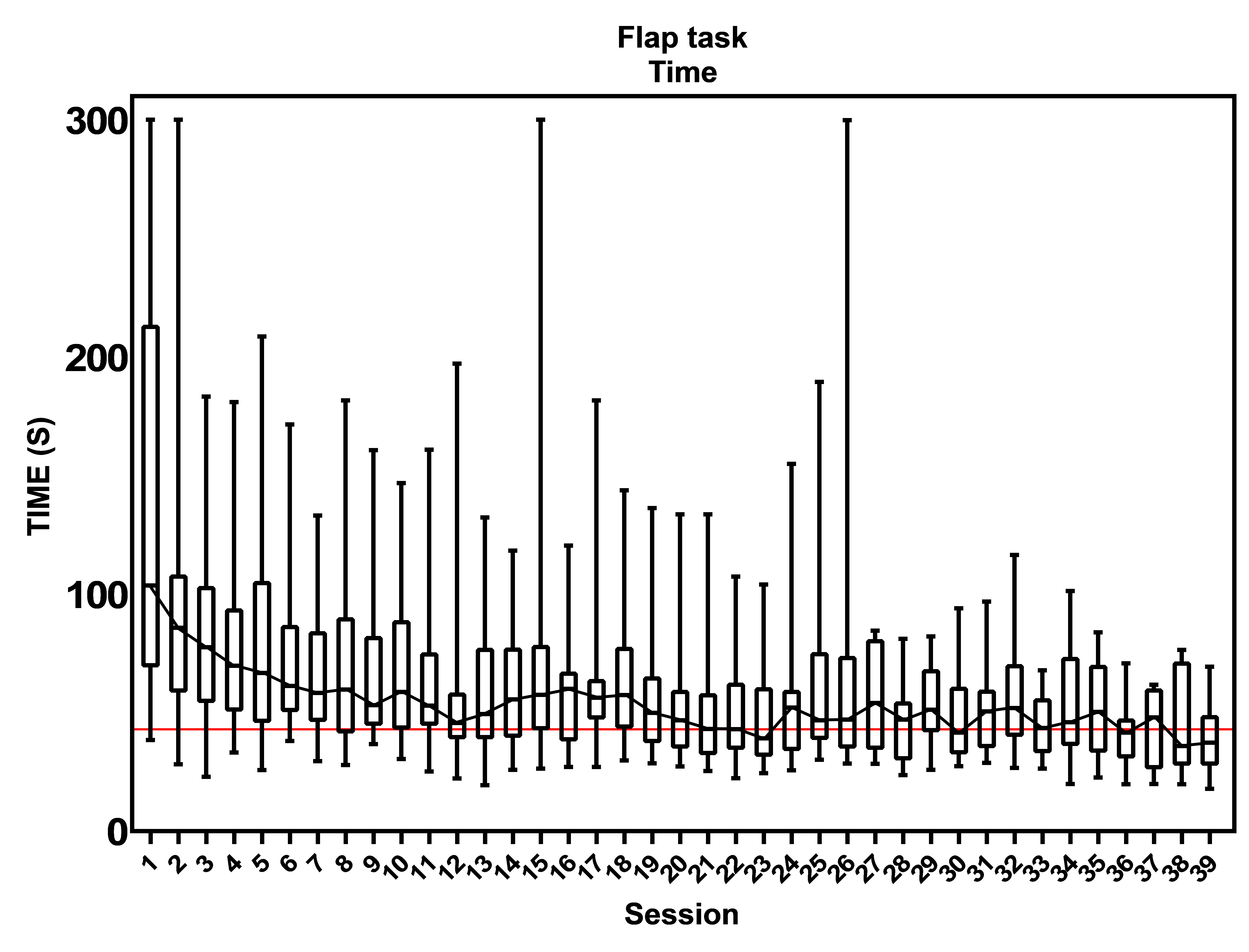


**Fig B3.** Proficiency graphs of task 3 “Flap task”.


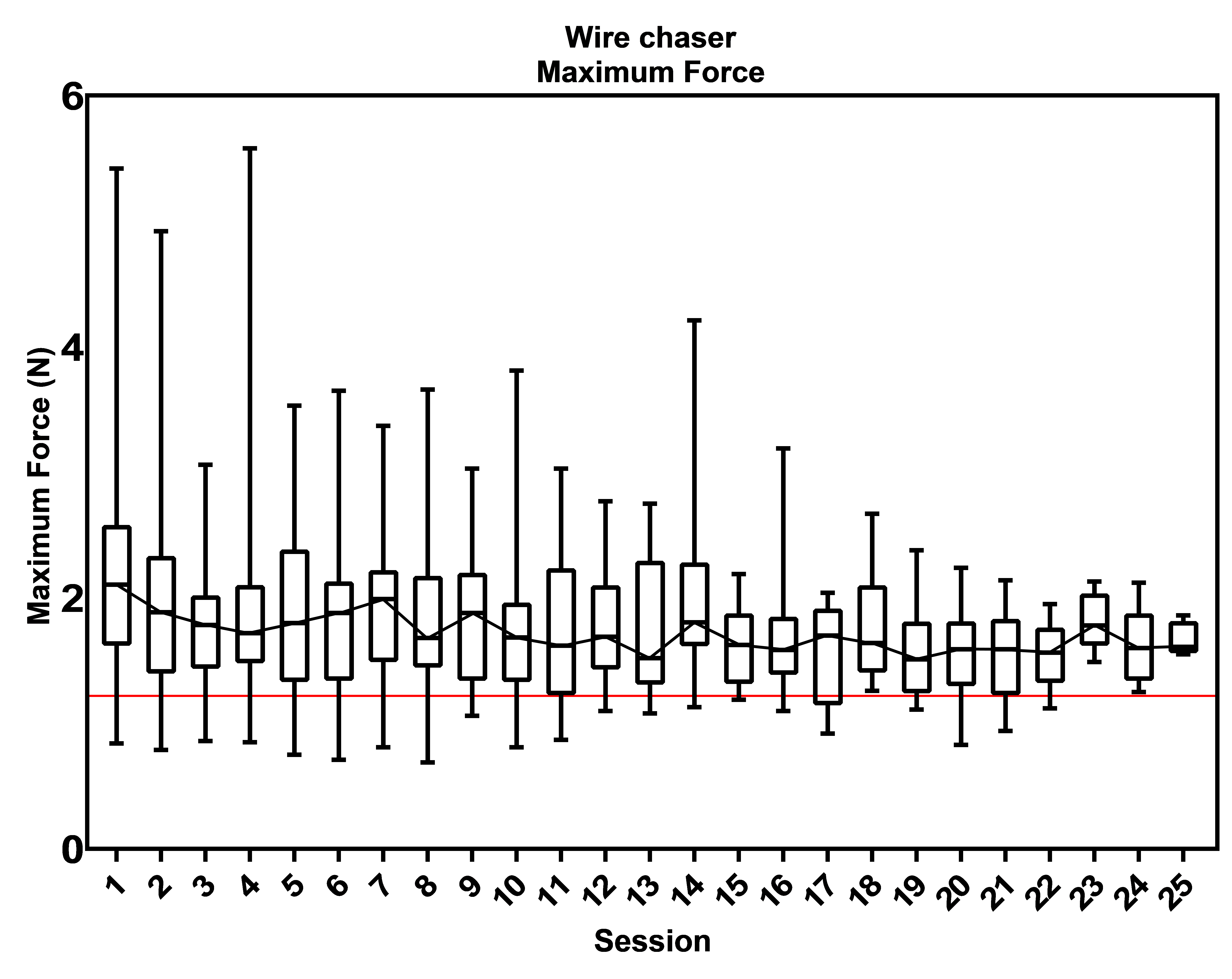


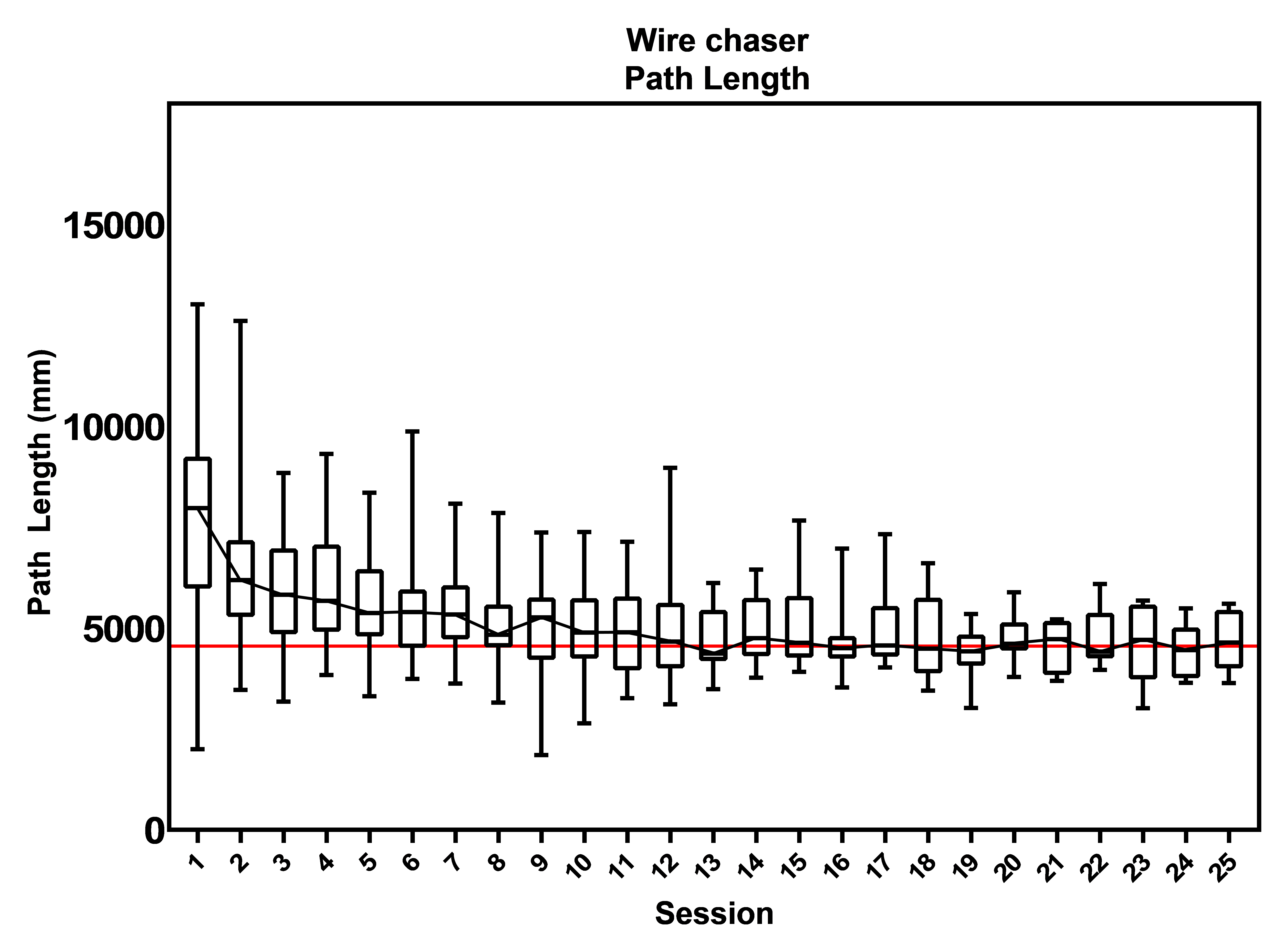


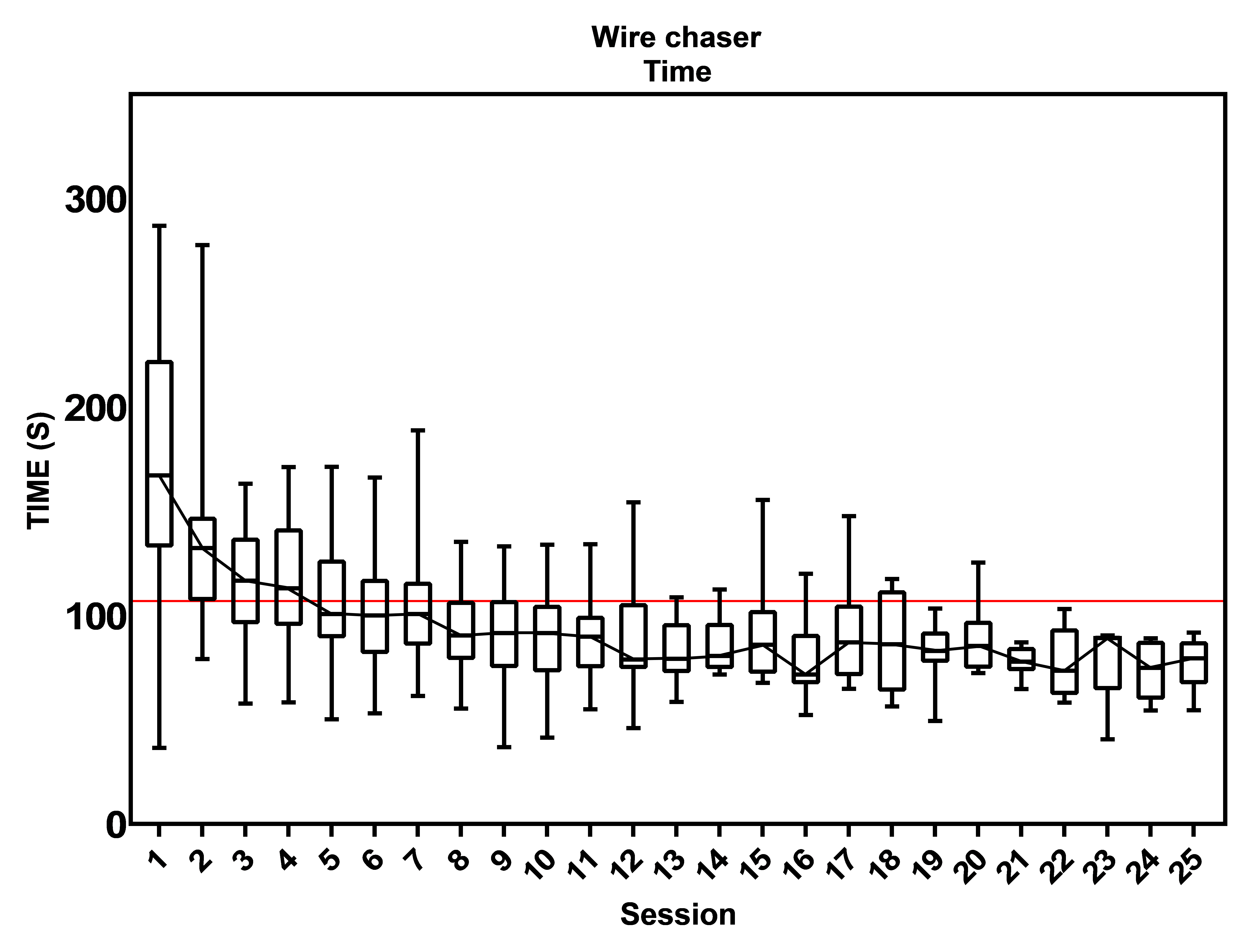


**Fig B4.** Proficiency graphs of task 4 “Wire chaser”.


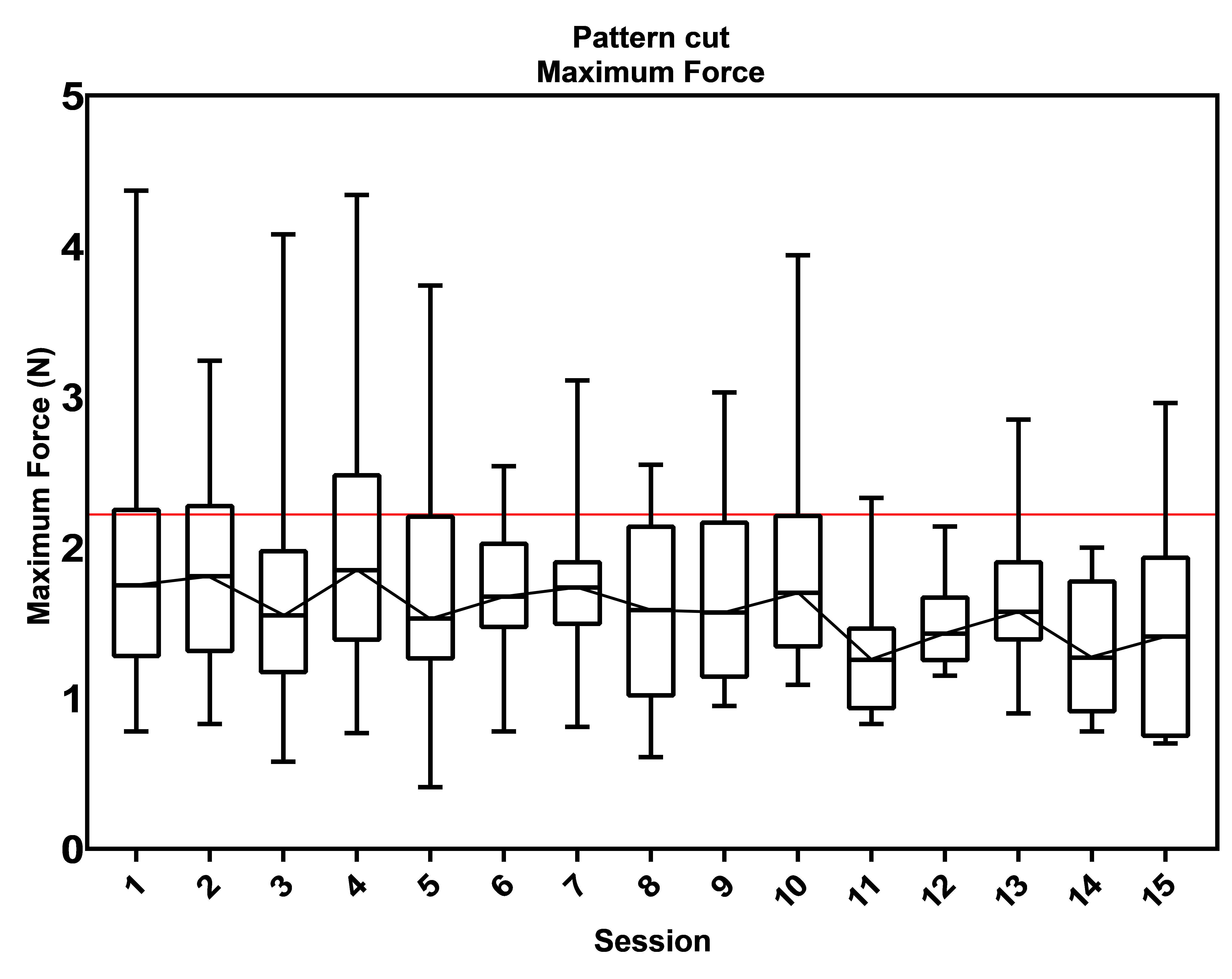


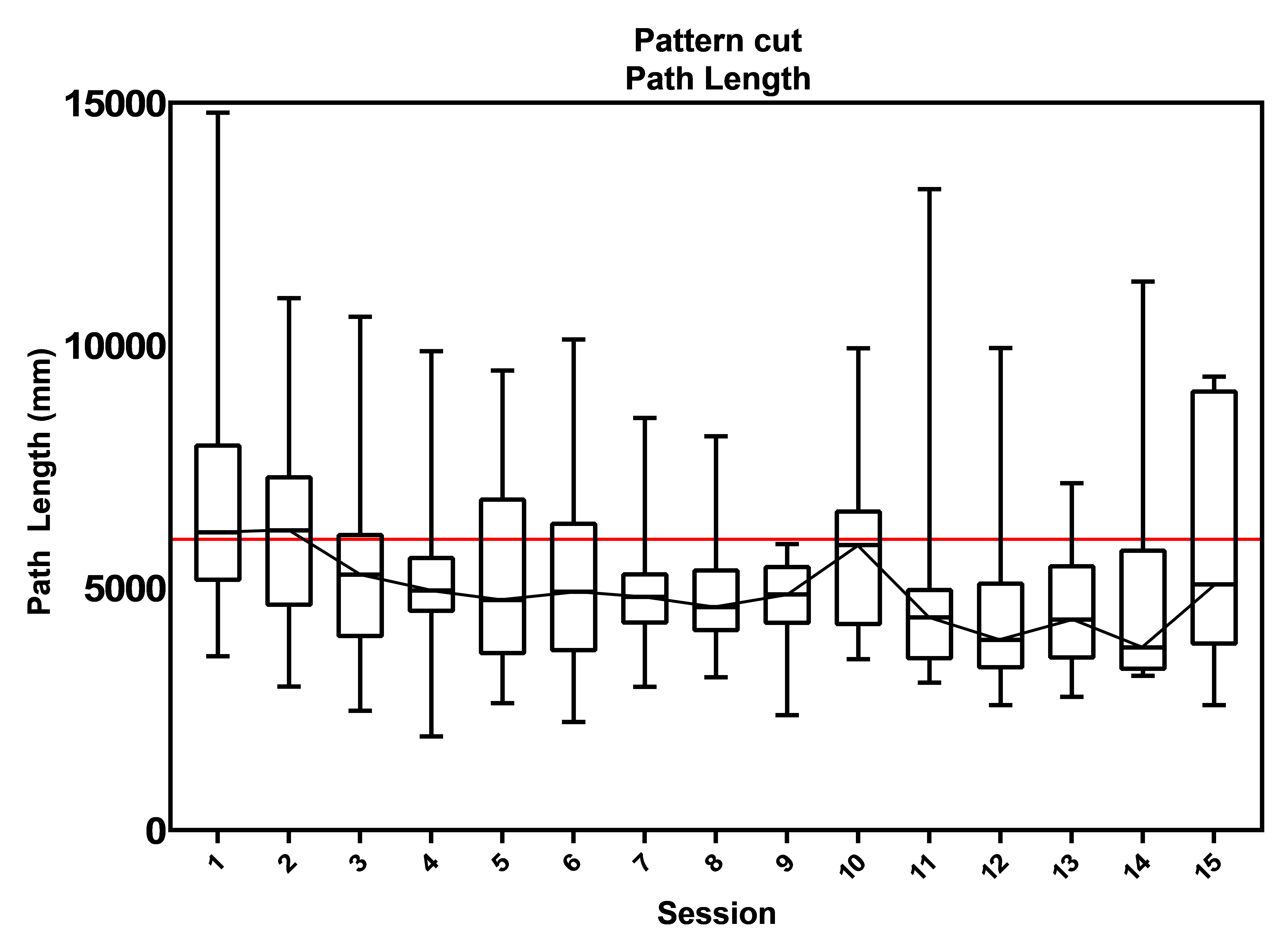


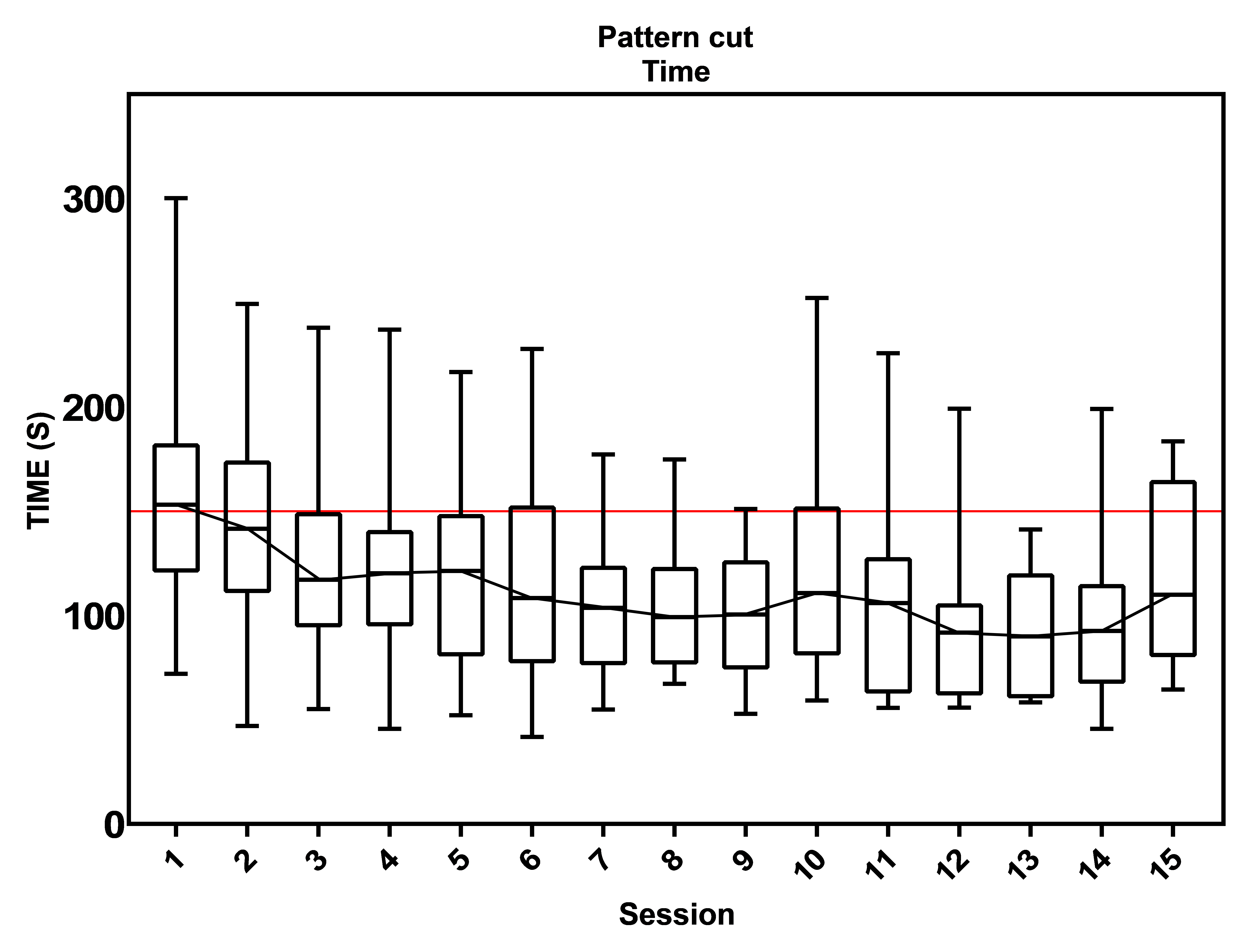


**Fig B5.** Proficiency graphs of task 5 “Pattern Cut”.


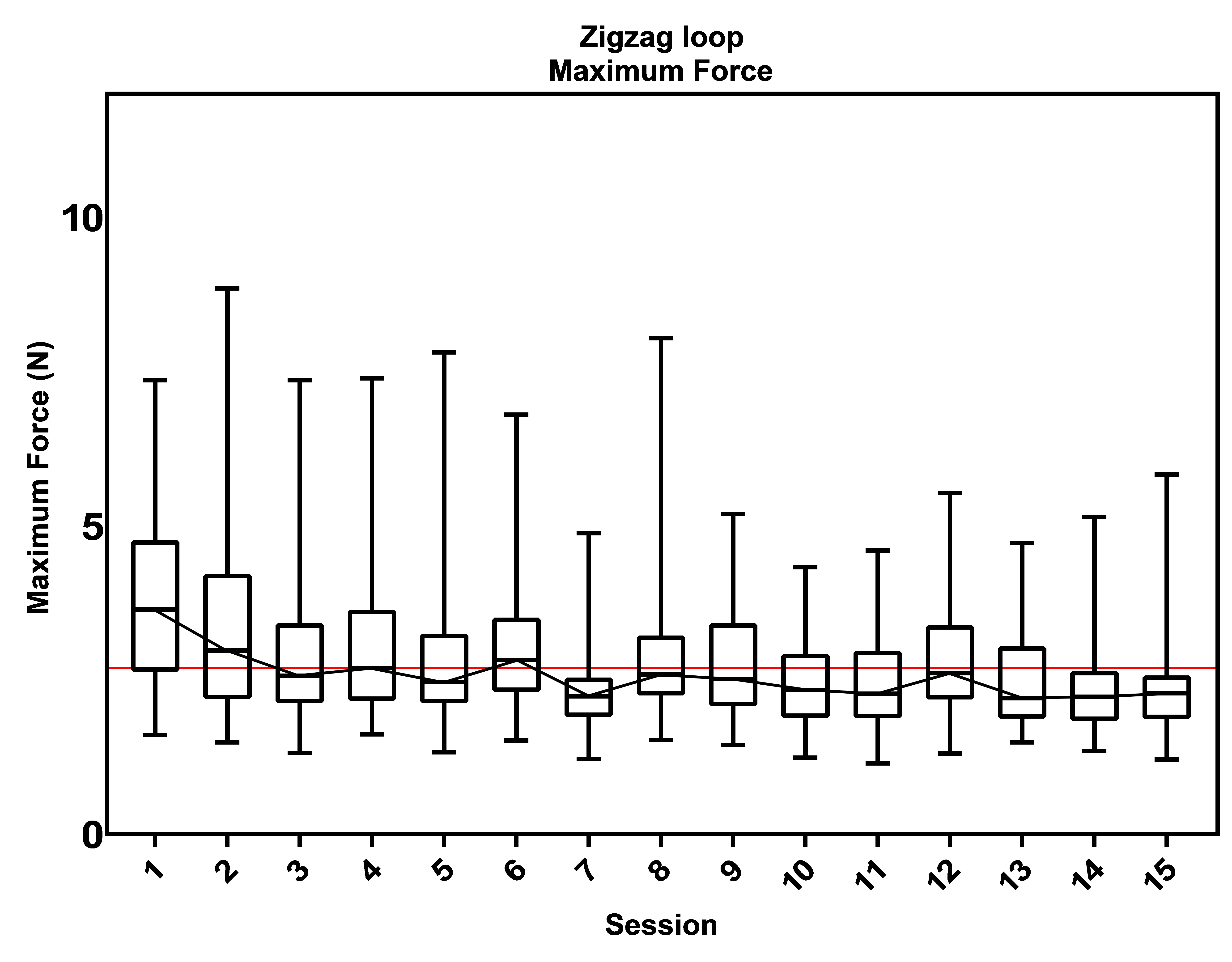


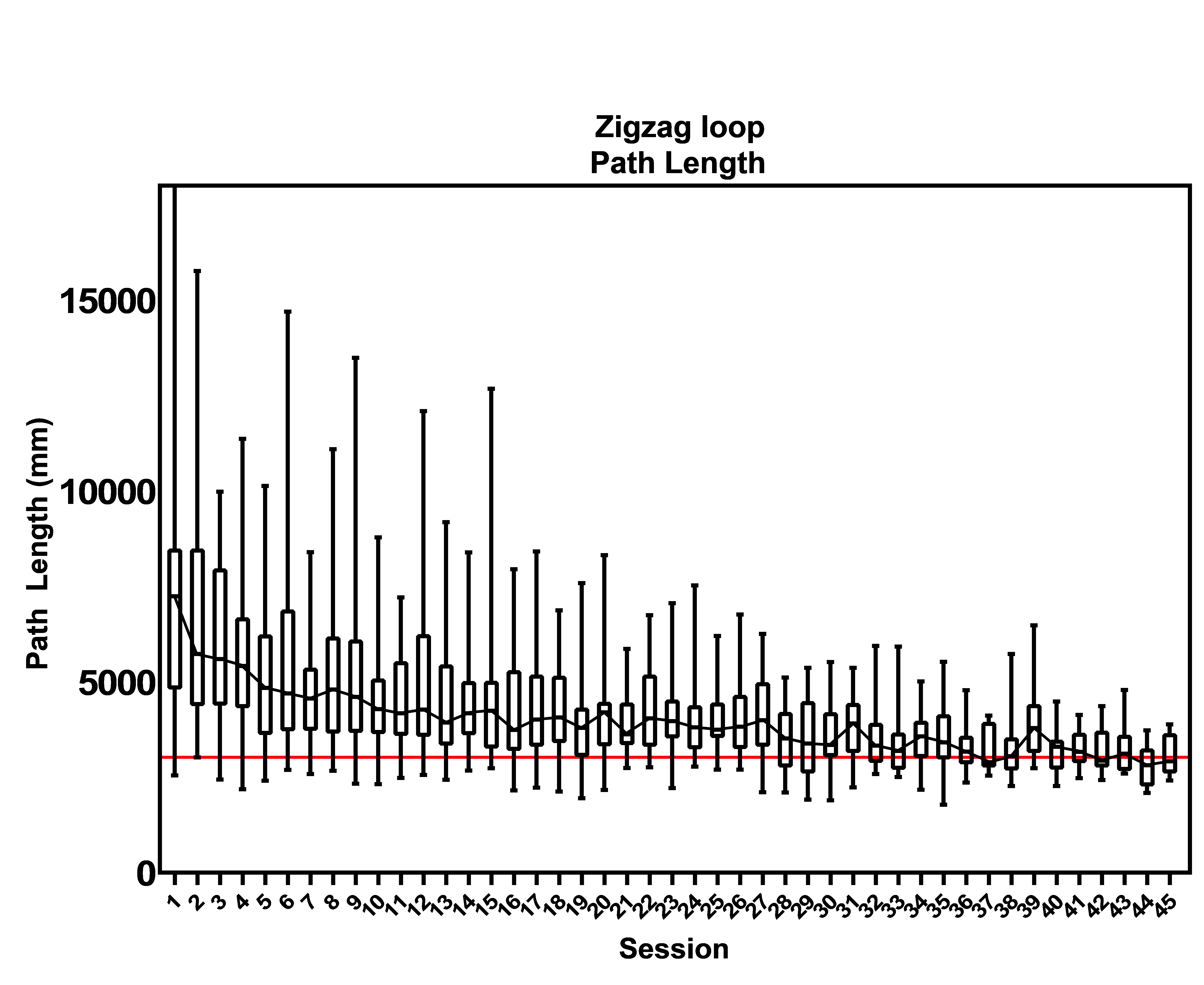


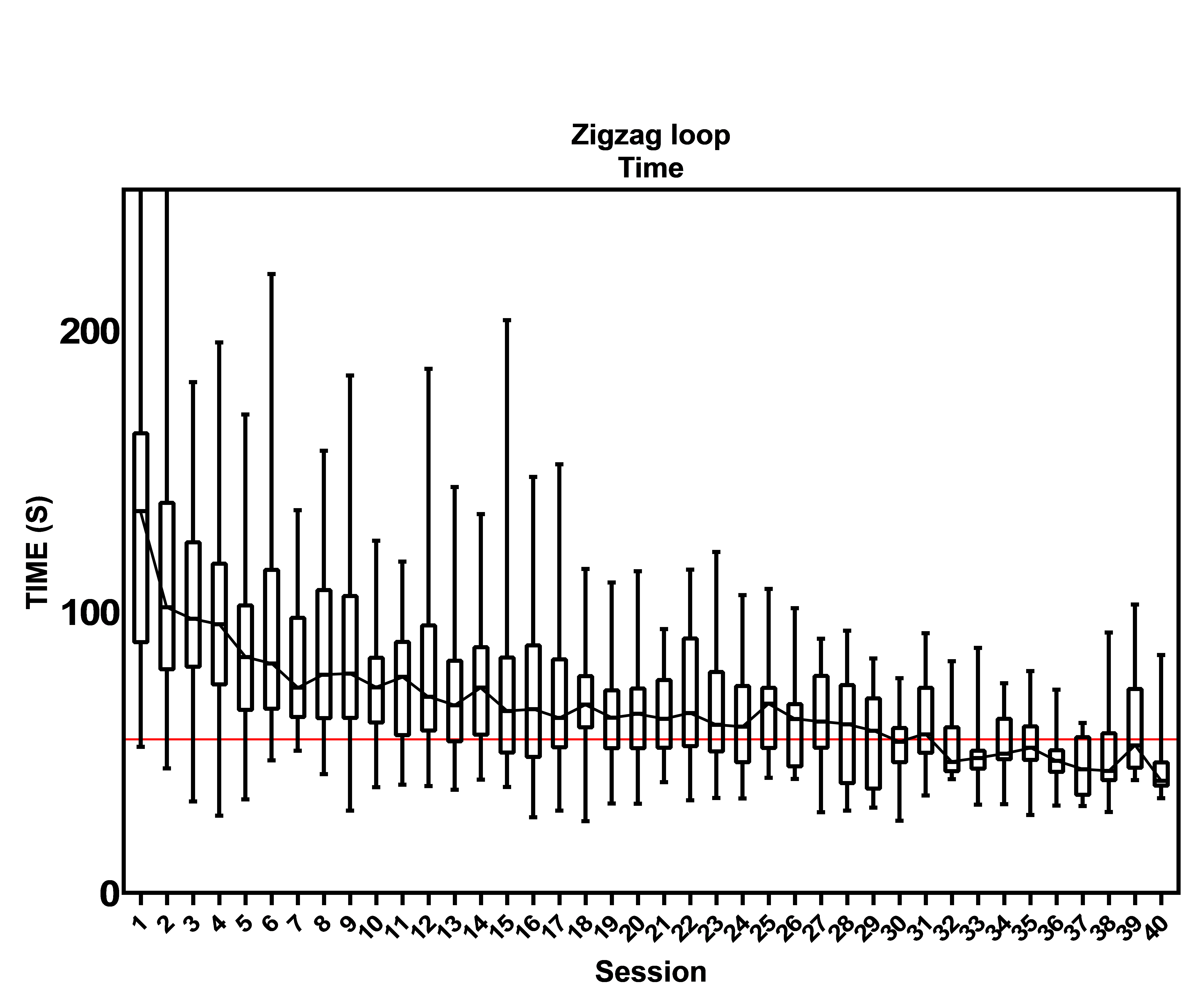


**Fig B6.** Proficiency graphs of task 6 “Zigzag loop”.

**Table B1**
*Mean session to proficiency and number of trainees that reached the proficiency level*

| Task | Benchmark | Mean session to reach proficiency | Number of trainees that reach proficiency |
| --- | --- | --- | --- |
| Post and sleeve |  |  |  |
| Time (s)  Path length (mm)  Maximum force (N) | 98.4 s  4810 mm  2.00 N | 21  32  4 | 38 / 42  37 / 42  42 / 42 |
| Loops and wire |  |  |  |
| Time (s)  Path length (mm)  Maximum force (N) | 86.0 s  3300 mm  3.01 N | 18  35  2 | 38 / 42  19 / 42  42 / 42 |
| Flap task |  |  |  |
| Time (s)  Path length (mm)  Maximum force (N) | 42.8 s  1993 mm  1.56 N | 36  36  2 | 36 / 42  37 / 42  42 / 42 |
| Wire chaser |  |  |  |
| Time (s)  Path length (mm)  Maximum force (N) | 106.6 s  4558 mm  1.22 N | 6  19  38 | 40 / 42  37 / 42  21 / 42 |
| Pattern cut |  |  |  |
| Time (s)  Path length (mm)  Maximum force (N) | 150.1 s  6000 mm  2.22 N | 3  3  1 | 42 / 42  42 / 42  40 / 42 |
| Zig-zag loop |  |  |  |
| Time (s)  Path length (mm)  Maximum force (N) | 54.6 s  3027 mm  2.70 N | 30  44  7 | 35 / 42  31 / 42  41 / 42 |

*Note.* Mean session at which trainees reach the proficiency level is determined by proficiency graphs (Fig 1 and Supplementary Fig 2–6).

**Table B2**
*Results of Linear Regression Analyses: Baseline performances as a predictor of the number of sessions needed to reach the benchmark of the parameters time, path length and maximum force*

| Post and sleeve |  | B | SE | t | Sig. (p) | 95% CI |
| --- | --- | --- | --- | --- | --- | --- |
| Time  Path length**  Maximum force** | Constant  X  Constant  X  Constant  X | -9.188  0.120  -0.166  1.29 E-4  0.000  0.188 | 3.238  0.018  0.175  0.000  0.093  .040 | -2.387  6.801  -0.951  5.823  -0.005  4.646 | 0.007 *  **0.000 ***  0.348  **0.000 ***  0.996  **0.000 *** | [-15.756, -2.621]  [0.085, 0.156]  [-0.521, 0.189]  [0.000, 0.000]  [-0.190, 0.189]  [0.106, 0.270] |
| Loops and wire |  | B | SE | t | Sig. (p) | 95% CI |
| Time ***  Path length **  Maximum force ** | Constant  X  Constant  X  Constant  X | 0.602  0.016  0.705  5.38 E -5  -0.396  0.206 | 0.630  0.004  0.375  0.000  0.087  0.028 | 0.955  3.588  1.882  0.860  -4.579  7.483 | 0.346  **0.001 ***  0.077  **0.402 n.s.**  0.000  **0.000 *** | [-0.676, 1.881]  [0.007, 0.025]  [-0.085, 1.496]  [0.000, 0.000]  [-0.571, -0.22]  [0.150, 0.261] |
| Flap task |  | B | SE | t | Sig. (p) | 95% CI |
| Time **  Path length **  Maximum force ** | Constant  X  X2 ****  Constant  X  Constant  X | -0.106  0.015  -4.21 E-5  0.230  1.12 E-4  -0.313  0.360 | 0.318  .005  0.000  0.120  0.000  .070  0.048 | -0.335  2.684  -2.034  1.915  4.718  -4.470  7.565 | 0.740  **0.011 ***  **0.050 ***  0.064  **0.000 ***  0.000  **0.000 *** | [-0.753, 0.541]  [0.004, 0.026]  [0.000, 0.000]  [-0.014, 0.474]  [0.000, 0.000]  [-0.455, -0.171]  [0.263, 0,456] |
| Wire chaser |  | B | SE | t | Sig. (p) | 95% CI |
| Time **  Path length **  Maximum force ** | Constant  X  Constant  X  X2 ****  Constant  X | -0.337  0.006  -2.245  0.001  -3.94 E-8  -0.707  -0.764 | 0.162  0.001  0.754  0.000  0.000  0.344  0.193 | -2.084  5.334  -2.988  3.245  -2.507  -2.054  3.967 | 0.044  **0.000 ***  0.005  **0.003 ***  **0.017 ***  0.054  **0.001 *** | [-0.665, -0.009]  [0.004, 0.008]  [-3.789, -0.719]  [0.000, 0.001]  [0.000, 0.000]  [-1.428, 0.14]  [0.361, 1.167] |
| Pattern cut |  | B | SE | t | Sig. (p) | 95% CI |
| Time **  Path length **  Maximum force ** | Constant  X  Constant  X  Constant  X | -0.508  0.005  -0.514  1.13 E-4  -0.292  0.206 | 0.111  0.001  0.115  0.000  0.071  0.039 | -4.571  7.231  -4.466  7.392  -4.080  5.348 | 0.000  **0.000 ***  0.000  **0.000 ***  0.000  **0.000 *** | [-0.733, -0.282]  [0.004, 0.007]  [-0.747, -0.280]  [0.000, 0.000]  [-0.437, -0.146]  [0.128, 0.285] |
| Zig-zag loop |  | B | SE | t | Sig. (p) |  |
| Time  Path length  Maximum force ** | Constant  X  Constant  X  Constant  X | 4.656  0.076  1.712  0.002  -0.189  0.155 | 4.345  0.034  5.212  0.001  0.144  0.041 | 1.072  2.239  0.329  2.760  -1.319  3.809 | 0.292  **0.032 ***  0.745  **0.010 ***  0.195  **0.000 *** | [-4.183, 13.495]  [0.007, 0.145]  [-8.964, 12.389]  [0.001, 0.004]  [-0.480, 0.101]  [0.073, 0.238] |

*Note.* Estimates are unstandardized coefficients. n.s. = not significant; * = p ≤ 0.05; ** = Log-transformed (Log10) dependent variable; *** = Square-root-transformed dependent variable; **** = Squared independent variable

**Table B3**
*Learning Curve Prediction: Linear regression equations. Y= Number of sessions needed to reach the benchmark; X= Baseline performances of the parameters time, path length and maximum force*

| Task | Untransformed data | Transformed data |
| --- | --- | --- |
| Post and sleeve |  |  |
| Time  Path length  Maximum force | Y = -9.188 + 0.120 * X | Log10 (Y) = -0.166 + 1.29 E-4 * X  Log10 (Y) = 0.000 + 0.118 * X |
| Loops and wire |  |  |
| Time  Path length  Maximum force | n.s. | SQRT (Y) = 0.602 + 0.016 * X  Log10 (Y) = -0.396 + 0.206 * X |
| Flap task |  |  |
| Time  Path length  Maximum force |  | Log10 (Y) = -0.106 + 0.015 * X – (-4.21 E-5) * X2  Log10 (Y) = 0.230 + 1.12 E-4 * X  Log10 (Y) = -0.313 + 0.360 * X |
| Wire chaser |  |  |
| Time  Path length  Maximum force |  | Log10 (Y) = -0.337 + 0.006 * X  Log10 (Y) = -2.245 + 0.001 * X – (-3.94 E-8) * X2  Log10 (Y) = -0.707 + -0.764 * X |
| Pattern Cut |  |  |
| Time  Path length  Maximum force |  | Log10 (Y) = -0.508 + 0.005 * X  Log10 (Y) = -0.514 + 1.13 E-4 * X  Log10 (Y) = -0.292 + 0.206 * X |
| Zig-zag loop |  |  |
| Time  Path length  Maximum force | Y = 4.656 + x * 0.076  Y = 1.712 + x * 0.002 | Log10 (Y) = -0.189 + 0.155 * X |

*Note. Estimates are unstandardized coefficients. Transformed models are either Log – Linear (Log10) or Square-root – Linear. n.s. = not significant*
